# New Method of Obtaining Concentrated Vinegar

**Published:** 1804-08-01

**Authors:** 


### New Method of obtaining Concentrated Vinegar. By Mr. Krueger.

Take red sulphat of iron, 3 ounces; acetit of lead, half an ounce. Mix them, and put them into a retort with a long neck; and having given a strong fire, two drachms of a very concentrated vinegar will pass over into the receiver. This acetic acid is quite pure, and has neither a sulphurous smell, nor does it contain sulphuric acid or lead. It is perfectly similar to that prepared after Westendorf's and Louitz's method.

---

### New Mode of making Lac Sulphuris.

Eight parts of sulphat of potash are ignited in a crucible with one part of charcoal powder; and after the mass becomes liquid, it is dissolved in four times its weight of water, and boiled; during which time sulphur is added till it can be no more dissolved. The mixture is afterwards diluted with twenty parts of water, and suffered to stand. Then it is decanted, and precipitated by diluted sulphuric acid. The quantity of this production amounts to one-half of the sulphat of pot-ash which has been employed. The sulphur may be also precipitated by concentrated vinegar, in order to obtain, at the same time, acetite of pot ash.

